# Widening the reach of family-based interventions for Anorexia Nervosa: autism-adaptations for children and adolescents

**DOI:** 10.1186/s40337-021-00511-8

**Published:** 2021-12-04

**Authors:** Rachel Loomes, Rachel Bryant-Waugh

**Affiliations:** 1 Maudsley Centre for Child and Adolescent Eating Disorders, Michael Rutter Centre, Maudsley Hospital, De Crespigny Park, London, UK; 2 Department of Child and Adolescent Psychiatry, Institute of Psychiatry, Psychology and Neuroscience, Kings College London, London, UK

**Keywords:** Anorexia Nervosa, Autism, Adolescence, Family-based therapy, Treatment

## Abstract

**Abstract:**

Family-based interventions are widely recommended as a first line treatment for children and young people with Anorexia Nervosa. There is clear evidence that model-adherent delivery of specific eating disorder focused family interventions has the potential to help adolescents with Anorexia Nervosa, who have typically engaged in extreme dietary restriction and lost a significant amount of weight over a relatively short period of time. Nevertheless, there remains a significant number of young people with restrictive eating disorders for whom family-based interventions for Anorexia Nervosa prove less effective, suggesting adaptations may be indicated for some. In this paper we provide a rationale and structure for considering a number of possible adaptations to the delivery of family-based therapy for anorexia nervosa specifically intended to enhance its relevance and potential effectiveness for children and adolescents on the autism spectrum; a subgroup known to represent a significant minority in eating disorder populations who have been identified as having relatively poor outcomes.

**Plain English summary:**

Past research has shown that certain family-based treatments are effective for many children and adolescents who develop Anorexia Nervosa. At the same time this type of treatment approach in its current form does not work for everyone. Recent research has highlighted the overlap between anorexia and autism and the need for the development of adaptations to existing treatments to better meet the requirements of people on the autism spectrum who develop anorexia. With this in mind we propose a number of autism-related adaptations that could be made to family-based treatments for anorexia. We hope that these might be formally tested in the future to see if these adaptations improve outcomes for this group of individuals.

## Background

Family-based therapies are currently recommended as first line treatment for children and adolescents with Anorexia Nervosa (AN) [1–3]. The best evaluated and most widely used are Family Therapy for Anorexia Nervosa, otherwise referred to as FT-AN [4], and Family Based Therapy for Anorexia Nervosa, more commonly known as FBT [5]. Both of these family-based approaches include the following: (1) a clear focus on working with the family to help the child return to age-appropriate eating behaviours that lead to weight restoration. This is combined with a strong message that the family is seen as a resource and not viewed as the cause of the problem; (2) an expectation that the parents take a lead in managing their child’s eating in the early stages of treatment; (3) a shift to handing back responsibility for eating to the child in an age appropriate manner once the risk related to weight has reduced; (4) in the later stages of treatment, the therapist broadens the focus from eating to adolescent and family developmental life cycle issues, to ensure that life is no longer organised by eating.

There is clear evidence that family-based therapies can help young people who have engaged in dietary restriction and lost a significant amount of weight over a relatively short period of time [6–8]. Whilst this approach works well for many, there remains a considerable number of young people who do not respond to the model as set out in the respective manuals, as evidenced by reported recovery rates of around 40–50% [9]. Research exploring moderators of treatment response in family-based treatments have indicated that obsessive–compulsive traits, maladaptive perfectionism, and high parental expressed emotion are predictors of a poorer response [10–12]. To date, the primary treatment adaptation that has been empirically investigated addresses the impact of parental expressed emotion on treatment outcome. This led to the recommendation that separated family-based therapies for AN be considered in families where there is a high degree of expressed emotion [11, 13]. It is also important to note, that as in many randomised controlled trials (RCTs), these family-based therapy models have primarily been developed and tested in samples without high levels of comorbidity. Further research is needed to explore whether adaptations to these models could provide increased benefits for those who do not respond to treatment in its current form.

One group highlighted as needing further consideration for treatment adaptations is people with AN who are autistic [14, 15]. Autism is a neurodevelopmental condition characterised by socio-communicative difficulties, presence of restricted interests and patterns of behaviour, and differences in sensory processing [16]. In line with current perspectives on language preferred by the majority of the autism community we predominantly use the terms ‘person on the autism spectrum’ and ‘autistic person’ [17, 18]. Over the past decade there has been increasing research and clinical interest in the overlap between autism and AN and the unmet needs of individuals, often women, who present with both [19–21]. Long-term outcomes for teenage-onset AN have been shown to be considerably worse if autism is also present [22]. Tchanturia and colleagues used the Autism Spectrum Quotient, short version (AQ-10) [23], which is a self-report measure of autistic traits such as attention to detail, attention switching, socio-communication and imagination. They found higher levels of autistic traits were associated with poorer treatment outcomes, more severe presentations, and longer inpatient admissions [24].

There are limited studies exploring the prevalence of comorbid autism and AN in adolescent populations [21]. One recent study reported a prevalence rate of 16.3% in a child and adolescent population of AN in Japan [25], although notably this figure relied on parent report questionnaires rather than the use of standardised assessment or screening tools such as the ADOS-2 [26] or the AQ-10. Adolescents with AN have been shown to have a higher number of autistic traits compared to control groups [27]. Further research is needed to better understand whether this reflects premorbid traits or whether it reflects the effects of restrictive eating, i.e. ‘state’ [28–30]. Either way an argument can be made for the need to consider how best to adapt assessment and treatment for children and young people (CYP) with AN who present with autistic features. This could be particularly valuable when there is a premorbid diagnosis of autism or a clear history of traits reported by the family prior to the onset of restrictive eating and weight loss. This need for adaptations is strengthened by reports from autistic adults with eating disorders and their carers highlighting their experiences of eating disorder services failing to adapt treatment [31]. In the UK, NICE guidelines state the need to adapt for autism [3], reflected in the recent development of the ‘PEACE pathway’ for adults with eating disorders and autism [32]. Adapting treatments for adolescents on the autism spectrum is also seen in other areas of mental health such as for obsessive compulsive disorder (OCD) [33].

In this paper we propose an approach that encourages systematic consideration of a number of potential adjustments to family-based interventions for AN with CYP with recognised autism or those displaying a significant number of autistic traits. Whilst FT-AN and FBT are conceptually similar, they do differ in several ways including how they structure the treatment and number of phases. In this paper we refer to the Eisler et al. [34] FT-AN treatment model and the four phases they set out. Similar adaptations could be applied to the equivalent phases of FBT. The core features of these interventions remain the same in the approach proposed here, but with particular consideration given to characteristics known to be more prevalent in autism. The four phases in FT-AN treatment are:

- Phase 1—engagement and development of the therapeutic alliance
- Phase 2—helping families to manage the eating disorder symptoms
- Phase 3—exploring issues of individual and family development
- Phase 4—ending treatment and discussion of future plans

### Proposed adjustments to FT-AN

In this section we discuss each phase in order, with Table 1 summarising proposed adaptations related to four core areas of autism that have been previously studied in adult eating disorder populations: (1) sensory considerations, (2) cognitive and behavioural rigidity, (3) difficulties with social interactions and relationships, (4) difficulties with emotional understanding, expression and regulation. Whilst several studies have explored the presence of these differences in AN populations generally [21], only a few have explored associations between these characteristics and autism/autistic traits within AN populations, such as thinking style and problems with social interactions [35] as well as reduced emotional expression [36]. More recently, Brede et al. [37] conducted a series of qualitative semi-structured interviews to better understand the development and maintenance of AN in people with an autism diagnosis. Perspectives were gathered from three groups: (1) autistic women with past or current experience of AN; (2) parents of autistic women with AN; (3) healthcare professionals from within the eating disorder and/or autism field. From this they put forward a model to highlight several areas of autism related difficulties that can directly or indirectly increase the likelihood of turning to harmful restrictive eating behaviours (Table 1). These areas map closely onto the four core domains we refer to in Table 2.

**Table 1:** Areas of autism-related difficulty adapted from Brede et al. [37] proposed model of autism-specific mechanism underlying restrictive eating difficulties

Area of autism-related difficulty	
Food specific sensory sensitivities	
Interoceptive awareness related to eating, digestion and bodily changes	
General sensory sensitivities	
Social interaction and relationships	
Understanding and regulating emotions	
Intolerance of uncertainty	
Rigidity and routine based behaviours	
Black-and-white, literal thinking around food, weight and eating	
Intense interests related to food, weight and exercise

**Table 2:** Summary of proposed autism-related adaptations for Family Therapy for Anorexia Nervosa (FT-AN)

	Phase 1 Assessment and engagement	Phase 2 Parent facilitated weight restoration	Phase 3 Handing back age appropriate responsibility and exploring issues of individual and family development	Phase 4 Ending well and relapse prevention
Sensory considerations	Assessment of sensory stimuli (e.g. noise, smell, light) that may impact on attention and focus (e.g. affecting eating or ability to participate in the session) as well as specific sensory sensitivities and preferences in relation to food	Ask parents to consider preferred foods prior to onset of AN cognitions as autistic characteristics linked to sensory differences may mean there have always been certain foods they avoid May need an adapted meal plan	May be ongoing struggles with eating in public due to sensory differences and may need ongoing adaptation at school	If sensory assessment tools have been used during assessment include these in relapse prevention/staying well plan
Cognitive and Behavioural rigidity	Consideration of environment and how family are invited to the assessment. Is there a way to increase aspects of certainty for the young person Using special interests to promote engagement and understanding	Awareness that a strong preference for routine and sameness may lend itself to young person benefitting from a predictable range of foods and eating routine during weight restoration Keeping the therapeutic process predictable and consistent	Introduction of safe certainty vs safe uncertainty and visual aid to help facilitate transition to phase 3 Managing expectations with regards to flexibility of eating Taking smaller, concrete, planned steps towards introducing increased flexibility with eating	Managing expectations from the start of therapy around when ending is appropriate Planning endings in advance and trying to avoid the ending coinciding with other significant life changes (e.g. starting a new school)
Difficulties with social communication and relationships	Use shorter sentences Avoid too many abstract ideas or idioms Be mindful that a lack of or unusual display of social cues does not always signal non-engagement	Continuing to adopt a more literal style of communication and avoiding figurative language	Consider whether or not difficulties with social relationships and/or identity played a role in the development and maintenance of AN Awareness of proposed female phenotype of autism	Ensuring that relapse prevention plan is clear, concise and visual so that young person has concrete summary to return to as needed
Challenges with understanding, regulating and expressing emotions	Availability of objects and activities for young person to use to help regulate arousal levels during the appointment Awareness that answering questions about emotions may be difficult	Additional psychoeducation on emotions, with anxiety/fear/guilt being the key emotions to focus on during this phase. Particularly around mealtimes Increased time on emotion regulation strategies with a focus on distress tolerance skills	Consideration of introducing additional emotion literacy and regulation skills	Awareness that endings involve change and how change can be anxiety provoking. Discuss and normalise this with young person and family

#### Phase 1—engagement and development of the therapeutic alliance

The first phase of treatment focuses on engagement and the development of the therapeutic alliance with the family, including the young person with AN. The therapist’s role is to create a secure base for the family in line with ideas proposed by Byng-Hall [38]. There are often high levels of family anxiety at the start of treatment due to physical risk and varying degrees of motivation in the young person. A strong therapeutic alliance is established to enable parents to feel safe enough to carry out the later tasks of Phase 2.

##### Therapeutic environment

Consideration should be given to making the treatment environment welcoming for the young person, whether they are attending a clinic or via a virtual platform. This includes considering needs related to possible difficulties with managing uncertainty, any difficulties with reciprocal communication, and any sensory hyper/hypo sensitivities. Finding change and the unexpected overwhelming is common in autism and reducing this ahead of the initial meeting can help promote positive engagement with the child and family from the start. Clinicians could consider how they can increase aspects of certainty for the child, e.g. a website they can look at with pictures or videos of the building, assessment process and team members. Parents could be contacted ahead of the appointment to ask if there is anything that can help their child feel more comfortable at the appointment such as a quiet room, low lighting, an object that they can fiddle with or one that provides comfort. Teams can provide a range of objects and activities that help with regulating the child’s arousal levels which may be higher than usual when being asked to talk about themselves with new people in an unfamiliar setting or having to cope with unfamiliar sensory input.

##### The assessment

The assessment is an opportunity for therapists to engage in problem free talk with the child to see if they have what is often referred to as a “special” or intense interest in a particular area. This information can also be held in mind for phase 2 where special interests could be used as short-term motivators to help young people with eating more or used as a distraction/distress tolerance activity when they have to tolerate difficult emotions during the weight restoration phase. It may also be helpful to hold in mind that it has been proposed that females and males with autism have qualitatively different restricted interests, and the majority of individuals presenting for treatment with AN are female. Rudy Simone, an author with a diagnosis of Asperger’s Syndrome, has commented in her writing that women with autism have restricted obsessions and interests which are not as unusual in subject matter as those of their male counterparts [39]. This can be linked to research that has concluded that amongst children with autism, boys are more likely than girls to prefer ‘object’ oriented or ‘screen-related’ interests [40]. Females on the other hand, are hypothesised to have interests that are more relational in nature, where the core feature of the interests involve developing and maintaining relationships with others. These “others” could be animals; they could be real people including celebrities; they could be imaginary friends or fictional characters [41, 42].

With regards to socio-communication difficulties, if autism is known or suspected clinicians can hold in mind some of the following: use of language such as shorter sentences, concise wording and avoidance of too many abstract ideas or idioms. The young person may find it hard to engage in eye contact and this should not be seen as a sign of non-engagement. Similarly, a child may give very direct or honest feedback, answer very literally, or struggle to answer a question about their emotions.

For sensory sensitivities, it could be helpful to consider environmental stressors related to noise, lighting, and smell. These will vary between individuals and therefore it is important to ask and not assume. Sensory processing differences, as well as preferences, can be discussed with the family and young person in conversation at assessment. Young people may also find it helpful to have a self-report checklist or tool to help identify and communicate their sensory needs. One such tool has recently been piloted in inpatient eating disorder settings with AN and autism with promising feedback [43]. This tool consists of a set of likert scales asking an individual to rate where they are on each scale 0–10 with 0 indicating significant hyposensitivity and 10 indicating strong hypersensitivity. The scale allows individuals to communicate where they are on a number of sensory domains (taste, smell, vision, sound, touch, texture) and then this can be used to adjust and calibrate treatment to personal needs. For example someone who identifies as hypersensitive to sound may find loud treatment spaces and eating environments overwhelming.

#### Phase 2—Helping the family to manage eating disorder symptoms

The second phase focuses on parents being supported to temporarily adjust their parenting style in order to help their child eat and restore weight to a healthy place. Theoretically this draws on structural family therapy techniques to restore parental authority [44] at a time where the eating disorder can often lead to families being drawn into reorganising around the illness [45], as well as use of reframing [46] to view this as parental care for their child rather than “taking control”.

##### Sensory considerations

For CYP with autism there may be foods that they struggle to eat due to sensory sensitivities. This sensory based avoidance where young people are reluctant to try certain foods based on sensory properties such as taste, texture, smell or visual appearance is commonly seen in people on the autism spectrum [47, 48]. Avoidance may be driven by disgust due to hypersensitivity in certain sensory domains, which understandably leads to avoidance of trying new foods. For others, reduced novelty seeking and fear of the unknown can also lead to a reliance on a limited number of ‘safe’ foods. When this longstanding selective eating co-occurs with AN it can be challenging to tease apart what food avoidance is due to previous sensory based avoidance and which foods are now being avoided due to fear of weight gain. In these circumstances we would encourage clinicians to spend more time at the start of treatment asking parents to identify their child’s preferred foods prior to onset of AN cognitions, as characteristics linked to sensory differences may mean they have always avoided certain tastes and textures.

As well as considering sensory based avoidance due to sensory sensitivities, young people on the autism spectrum can also present with a very narrow range of preferred foods due to high levels of cognitive and behavioural rigidity which presents as a strong preference for routine and sameness. Thought will be needed with the family to discuss how much to change routine and to give consideration to the need to have a fairly consistent and similar set of foods every day if that helps the child to restore weight. Flexibility around variety can be explored once risk related to weight has reduced.

In some teams it is protocol or there is the option to give families a meal plan at the start of treatment. This can be helpful for families where parental confidence related to feeding their child has reduced since the onset of AN. It can also be helpful in reducing room for negotiation in the family. In our experience it is not unusual for a young person to be worried that their parent will give them more than they need and parents worry that their child will continually try and negotiate less food. Therefore in these situations a meal plan can ease anxiety for both the young person and the parents as it reduces the need for decision making at a time when emotional arousal around eating is heightened. If a service has a generic weight restoration meal plan or guide, this may not fit with a child on the autism spectrum due to their previously selective eating and narrow range of accepted foods. Here more thought is needed, with a dietitian where possible, to create a weight restoration meal plan that is based on the range of foods the child ate prior to the onset of AN cognitions.

As well as consideration of the sensory properties of food, it is also important to conduct a broader assessment of sensory differences that may impact eating (e.g. noise, smells). It can be helpful to explore with the family whether or not there are certain environments that the child is more likely to eat and does this fit in with family expectations around meals and eating. Similar thinking can be applied to eating at school in the early stages of treatment where canteens can be overwhelming for autistic individuals due to the noise, crowdedness and smells. Food can also be unpredictable for schools that serve hot meals. Adaptations can include identifying a separate quiet space to eat, having the school meal schedule in advance and being able to bring in a packed lunch of preferred foods.

##### Meal times

In the early stages of treatment in FT-AN, close monitoring and support from parents at mealtimes is seen as an important step to facilitating weight restoration and reducing risk related to low weight. In FT-AN, emphasis is given to helping parents acknowledge the anxiety their child is feeling due to fear of weight gain. There is emphasis on parents validating their child’s emotions at mealtimes whilst also remaining firm and encouraging them to eat. Additional psychoeducation may be needed in the early sessions to help both the young person and parent. Autism has been shown to have high heritability (64–91% in meta-analysis) [49]. Therapists benefit from being aware of this because it indicates that there is a reasonable likelihood that one or both parents of an AN patient on the autism spectrum may themselves be autistic or have an elevated number of features. This is important when we consider what we ask of parents in phase 2 of treatment, particularly at mealtimes when parents need to be able to validate their child’s anxiety which may present as high distress or aggressive behaviour. As autism is associated with impaired emotional regulation [50] parents who are on the spectrum themselves may struggle to recognise or effectively respond to their emotions. Therefore they may benefit from additional coaching in emotional literacy and regulation in order to have the skills and confidence to support their child with developing their own emotional awareness and use of adaptive coping strategies. Parents may also benefit from learning distress tolerance skills and emotion regulation strategies to cope with the impact of supporting their child at mealtimes. Both the FT-AN and FBT manuals detail key principles for supporting children at mealtimes, primarily the importance of consistency, firm and caring boundaries, as well as clarity around expectations.

#### Phase 3—Handing back responsibility to the young person and widening the focus

Phase 3 is moving towards the young person taking more responsibility for their eating appropriate to their stage of development and exploring issues of individual and family development. A strong therapeutic relationship and trust between the family and therapist is needed in order to enable the family to be open to working towards tolerating safe uncertainty [51].

**Fig. 1:**
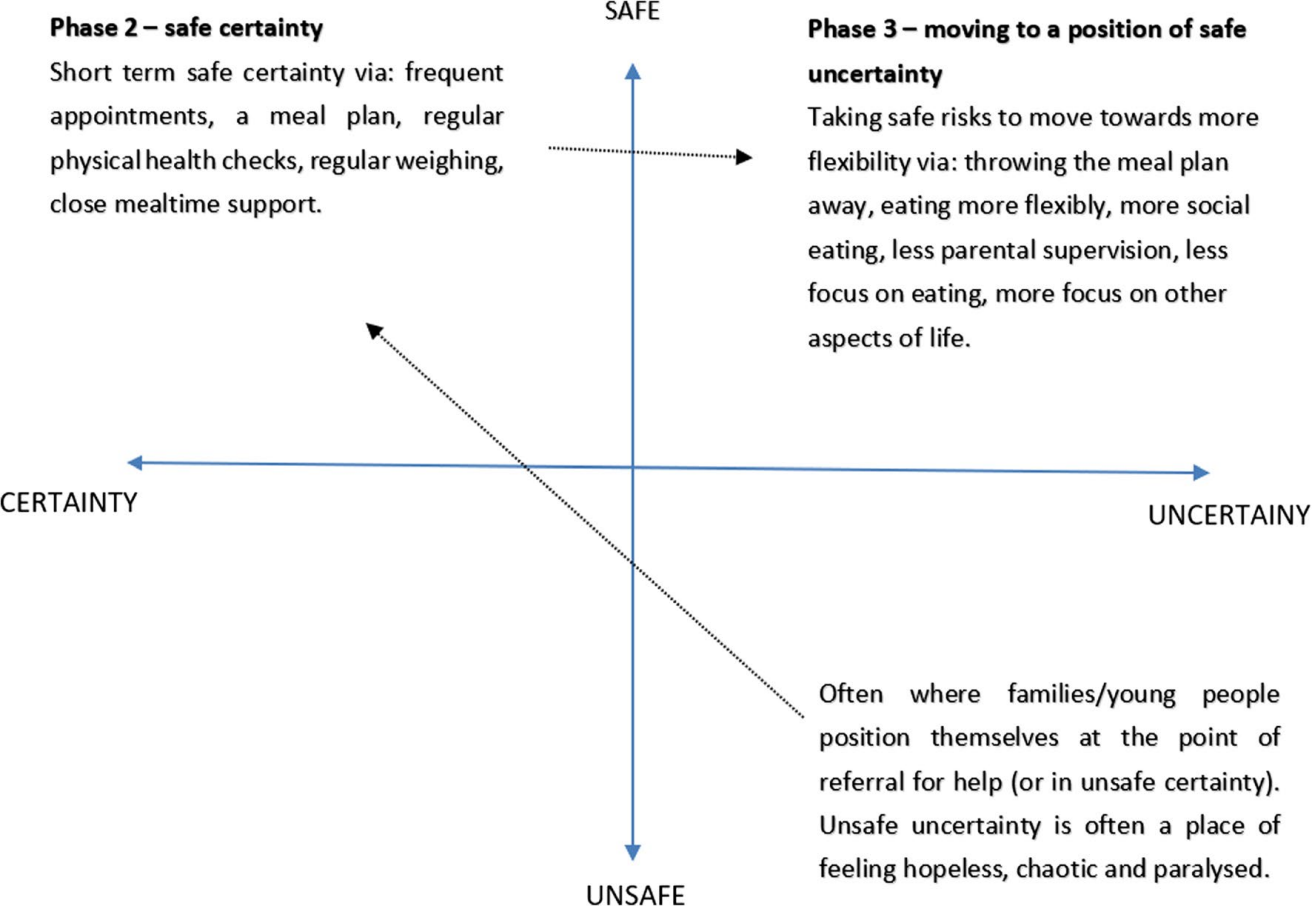
Towards a position of safe uncertainty—adapted from Mason [51]

In the FT-AN manual reference is made to Mason’s concept of moving from a position of “safe certainty” to “safe uncertainty” [51]. Using Mason’s original grid can be a helpful visual aid to guide the family through the rationale for the move from phase 2 to phase 3 of FT-AN and help the young person and family identify potential barriers to moving to a position of safe uncertainty (see Fig. 1). Mason introduced the idea of safe uncertainty as a position from which one can experiment with small differences repeatedly over time to create change [52]. He used a four-quadrant model to describe unsafe certainty, unsafe uncertainty, safe certainty and safe uncertainty and how people seeking therapy often start from a place of unsafe certainty or unsafe uncertainty, seeking safe certainty. Mason writes about how initially a family may need short term safe certainty in therapy in order to gain a sense of personal agency in their ability to be self—and other—protective. In phase 2 the clinician provides temporary safe certainty to the family through frequent appointments, a meal plan, regular physical health checks, regular weighing and close mealtime support. Phase 3 involves moving to a position of safe uncertainty by taking safe risks to move towards more flexibility to help the young person and their family navigate the uncertainties of life and avoid becoming stuck in the narrow and rigid patterns of behaviour that come from safe certainty. This includes letting go of the meal plan, more social eating, less parental supervision and focusing on areas of development outside of the eating. For CYP on the autism spectrum this can be an opportunity to identify in a family session how some of the known features of autism can make this transition harder, which can in turn help parents empathise more with why their child might find this change difficult. This can include autistic traits such as intolerance of uncertainty, inflexible thinking styles, preference for sameness and routine. Other, less obvious, autism related barriers might include letting go of the AN identity for those who are struggling to understand themselves and where they fit socially. Once these barriers are identified, the therapist can make a plan with the family around what might be most helpful in phase 3. The FT-AN manual sets out that this can be a time in treatment to consider individual sessions or adjunctive therapy work. The barriers could be explored within a family setting as set out in the original manual.

##### Expectations for change

For CYP with a restrictive eating disorder and co-occurring autism it will be important to consider what is realistic for the young person in terms of how flexible they are with their eating. This is because sensory sensitivities, low interest, or preference for sameness and routine may mean that their eating norm consists of a fairly narrow range of foods. This also links in with exploring with parents their expectations for change, especially if they are people who take pleasure from trying new foods and eating from a wide range of different tastes and textures. Additionally, in FT-AN, the transition to taking more responsibility for eating and increasing flexibility may be unwelcome, especially if they prefer routine and familiarity, or if they show low interest in food and find it hard to eat independently. These considerations should also be given to how food is eaten. Children with a higher preference for sameness for routine may prefer eating at similar times or have routines for how they like to eat, and discussion will need to be had with the family to explore any possible impacts of this, e.g. can the family agree with the young person that they keep routines where possible but that the young person is able to adjust these with advanced warning when they need to, e.g. holidays or special occasions.

##### Socio-communication challenges

For CYP with diagnosed autism or a high level of autistic traits, it will be important to consider with the young person and family about how socio-communication difficulties may impact their ability to take age-appropriate responsibility for their eating. For example, exploring with the child to see how they feel about friendships and school, and whether or not this is a barrier to them moving back to eating with peers if they had been eating with parents or a teacher during the weight restoration phase. When they are then expected to transition back to unsupervised eating, they may feel unmotivated to return to this if they were previously struggling with social or sensory aspects of the school environment.

In the adult eating disorder literature on AN and autism, women have described turning to restrictive eating as a way to “fit in” with peers or have an identity when they don’t feel ‘normal’ [37]. If this is identified by the young person as a function of their AN, this is when additional focus may be needed to help them explore issues around identity and relationships. In recent years there has been more focus on how females on the autism spectrum present differently to their male counterparts. This has been referred to as the “female phenotype of autism hypothesis” [53], Examples of gender differences noted in the research include autistic females reporting high use of camouflaging or masking [54, 55], having higher social interest and motivation [56] and being more likely to struggle with internalising difficulties such anxiety and depression [57]. In their model, Brede et al. [37] highlight unrecognised autism as a potential moderating factor for leaving women more vulnerable to turning towards restrictive eating as a way to manage unrecognised autism-related difficulties. We suggest that phase 3 of FT-AN is a good time to explore these areas with the young person and family. For those who demonstrate elevated autistic traits without a prior diagnosis this may also be an appropriate time to explore whether or not to refer on for specialist assessment. This is because at this point the young person will no longer be in a state of semi-starvation, which may have been mimicking autistic characteristics.

A number of young people on the autism spectrum display difficulties with emotional recognition, expression and regulation [58]. This is sometimes referred to in the literature as alexithymia [59]. Brede et al. [37] noted from their interviews that a number of women turned to restrictive eating to help them manage emotions that they did not understand or feel able to control. Some described how focusing on numbers and weight provided a sense of calm, whilst others noted the dampening effect starvation had on their arousal levels. Again, if this is identified by the young person as a barrier to them letting go of the eating disorder therapists should consider how to target this need through therapy. Further research on the most effective approach is needed. One option may be to explore emotional literacy and regulation in family sessions. For example, through the use of genograms as a visual aid to explore how other members of the family recognise and cope with different emotions, particularly during adolescence. The therapist may also consider an adjunctive piece of individual therapy that focuses on enhancing emotional literacy, expression and regulation.

#### Phase 4—Ending treatment and discussion of future plans

Phase 4 focuses on ending well and relapse prevention. At this stage in therapy the time between sessions is further increased to reflect the reduced reliance of the family on the therapist. The main adaptations here focus on supporting the young person with managing the change and uncertainty that comes with ending, as well as ensuring the use of visual aids to summarise the work done throughout treatment. Phase 4 is set out in the FT-AN manual in a way that helpfully accounts for some of the anticipated needs of someone with co-occurring autism. For example the manual emphasises the importance of having endings on the agenda from the first phase of treatment. This is done by laying out the roadmap for treatment and managing expectations from the start; that people are not expected to be ‘symptom free’ at discharge. Due to the additional difficulties with tolerating uncertainty, this is important to keep coming back to and also provide psychoeducation to the young person and family that it is normal to experience anxiety about endings. This can be explored with families in terms of managing change and uncertainty. The FT-AN manual also refers to relapse-prevention and as with previous suggestions this could be enhanced to include visual summaries. Depending on the identified needs of the young person this could include:

- A visual strengths and vulnerabilities based formulation or ‘passport’ of the young person and family. This can include autistic features and how these can be both strengths and also vulnerability factors for the young person.
- A visual relapse prevention document for the young person and parents to complete over the last few sessions.

The above can be kept and shared with future professionals, as well as being a document that the young person and family can refer to if they encounter setbacks. Linked to the ideas set out in this phase of treatment, this is also a way of trying to empower everyone in the family to acknowledge their role in the changes that have taken place over therapy, with less locus of change placed on the therapist or wider clinical team. Examples of such documents can be found at the Peace Pathway website in the resources section for patients. This website caters specifically to those with co-occurring eating disorders and autism and includes wellbeing communication passports and formulation worksheets [60]

## Conclusion

In this paper we have proposed a number of adaptations to FT-AN for those who either present with a diagnosis of autism or present with a high degree of autistic features. At this point these adaptations are presented at a conceptual level and via anecdotal clinical experience of their use in clinic with this population; they need further formal testing in a clinical setting to evaluate whether or not they can contribute to improved treatment outcomes. As the core tenets of family-based interventions for AN remain the same, it may be that clinicians could use a checklist to consider the relevance of the proposed adaptations for specific individuals. Following implementation as indicated, feedback from young people, parents and clinicians could contribute to determination of the acceptability, feasibility, and effect of adjustments made. In order to introduce consideration of adaptations in a systematic manner, appropriate identification of CYP who might benefit from an adapted approach is needed. Whilst it is straightforward to identify CYP with a pre-existing autism diagnosis, we know from recent qualitative research in adult populations that most women with AN receive an eating disorder diagnosis before their autism is formally recognised [15, 30]. Furthermore, eating disorder clinicians report low confidence when it comes to identifying and referring young people onward for an autism assessment [14]. This highlights an unmet need in eating disorder services to develop better pathways for detecting individuals who may benefit from an autism assessment. Research and opinion is mixed when it comes to the timing of having a conversation about suspected autism, especially because of the ongoing debate about to what degree the effects of starvation mimic autistic traits. We suggest that in most instances discussion with the young person and family ideally takes place once the child is no longer showing symptoms of being in a starved state. We advise against linking this to a specific number or median BMI centile; in our experience people who lose weight rapidly but remain in the ‘healthy’ weight range can be in a semi starved state. The reverse can also be true where some individuals function very well at a low weight, including for women, experiencing regular menses. In the context of FT-AN such a discussion may best be considered when individuals are in Phase 3 of the treatment and autistic characteristics are hypothesised to be maintaining ongoing difficulties for the individual that impede their ability to take age-appropriate responsibility for their eating and move away from the eating disorder. Screening tools, such as the adolescent AQ [23, 61], which comes in parent-report and adolescent-report, or the SRS-2 [62], are a way to introduce routine screening into assessments. Scores from these screening measures can then be used as part of the conversation with families about the rationale for exploring whether or not an assessment may be helpful.

A further consideration is whether similar adaptations could be applied to other restrictive eating disorders, such as Avoidant Restrictive Food Intake Disorder (ARFID). Family-based treatment for anorexia nervosa in adolescents has been adapted for ARFID [63–65] with case reports describing its use in the context of different drivers of food avoidance or restriction [66, 67]. Emerging research and anecdotal clinical experience highlights the overlap between ARFID and autism [68]. Therefore it may be helpful to consider how some of the adjustments outlined in this paper could helpfully be applied to those with ARFID where there is a need for weight gain.

## Data Availability

Not applicable.

## Abbreviations

AN: Anorexia Nervosa
CYP: Children and young people
FBT: Family Based Therapy for Anorexia Nervosa
FT-AN: Family Therapy for Anorexia Nervosa
RCT: Randomised Control Trial
